# SNPnexus: assessing the functional relevance of genetic variation to facilitate the promise of precision medicine

**DOI:** 10.1093/nar/gky399

**Published:** 2018-05-11

**Authors:** Abu Z Dayem Ullah, Jorge Oscanoa, Jun Wang, Ai Nagano, Nicholas R Lemoine, Claude Chelala

**Affiliations:** 1 Bioinformatics Unit, Centre for Molecular Oncology, Barts Cancer Institute, Queen Mary University of London, London EC1M 6BQ, UK; 2 Centre for Molecular Oncology, Barts Cancer Institute, Queen Mary University of London, London EC1M 6BQ, UK; 3 Centre for Computational Biology, Life Sciences Initiative, Queen Mary University of London, London EC1M 6BQ, UK

## Abstract

Broader functional annotation of genetic variation is a valuable means for prioritising phenotypically-important variants in further disease studies and large-scale genotyping projects. We developed SNPnexus to meet this need by assessing the potential significance of known and novel SNPs on the major transcriptome, proteome, regulatory and structural variation models. Since its previous release in 2012, we have made significant improvements to the annotation categories and updated the query and data viewing systems. The most notable changes include broader functional annotation of noncoding variants and expanding annotations to the most recent human genome assembly GRCh38/hg38. SNPnexus has now integrated rich resources from ENCODE and Roadmap Epigenomics Consortium to map and annotate the noncoding variants onto different classes of regulatory regions and noncoding RNAs as well as providing their predicted functional impact from eight popular non-coding variant scoring algorithms and computational methods. A novel functionality offered now is the support for neo-epitope predictions from leading tools to facilitate its use in immunotherapeutic applications. These updates to SNPnexus are in preparation for its future expansion towards a fully comprehensive computational workflow for disease-associated variant prioritization from sequencing data, placing its users at the forefront of translational research. SNPnexus is freely available at http://www.snp-nexus.org.

## INTRODUCTION

The application of sequencing technologies to research and clinical settings has increased dramatically, generating vast amounts of data about variations in our genomes that could explain some differences in disease susceptibility, progression and how patients react to drugs. However, the analytical strategies to interpret this data and translate *in-silico* findings into clinical applications remain a major challenge in research. SNPnexus is a popular analytical tool that was designed to meet this need (1,2,3). It allows researchers to assess the potential significance of candidate sequence variants and points to the altered gene/protein isoforms that may lead to phenotypic changes, thereby guiding future experimentation.

SNPnexus has already proven itself as a pioneer in providing the most elaborate set of annotations for a diverse range of sequence variation data. Since its last release in 2012, the positive feedback from the ever-growing national and international SNPnexus user community has encouraged us to improve our system with new and more advanced annotation features. Here, we present the current release of SNPnexus, which has the whole range of annotation categories for the latest human genome assembly, capable of providing detailed annotation for coding and noncoding variations alike, can identify potential therapeutic targets and is equipped with an improved query and output interface. With these additions and improvements, we ensure that SNPnexus remains at the forefront of research for broadening our understanding of the functional role of genetic variation and their impact on health and disease.

## NEW DEVELOPMENTS

**Figure 1.**
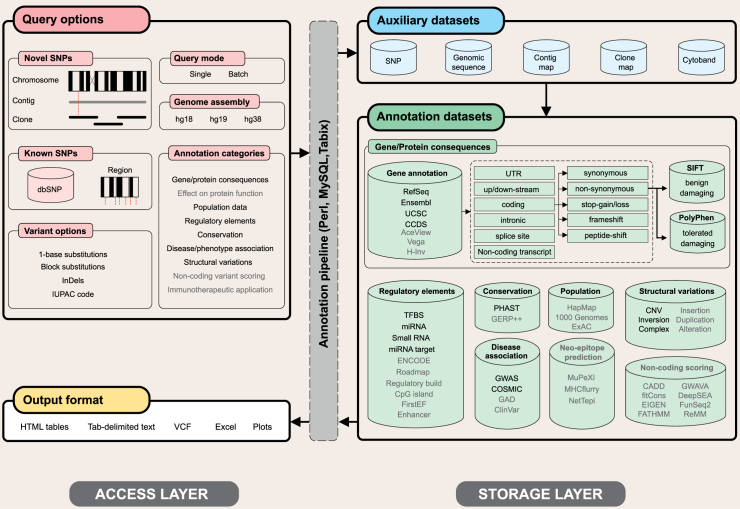
A schematic overview of the request-response architecture of SNPnexus. The annotation features coloured in grey are only available when selecting certain genome assemblies.

The basic request-response architecture of SNPnexus has been enhanced. A Perl pipeline connects to the MySQL tables and annotates the submitted data on the fly. Tabix (4) queries are used to process VCF/BED files. For each variant, in addition to the requested annotations, SNPnexus provides cytogenetic and physical mapping on chromosome/contig, indicates whether the variant is in dbSNP (5) and provides information on overlapping or neighbouring up/down-stream genes. A snapshot of the complete features of SNPnexus is presented in Figure 1.

### Expansion of annotation categories

#### New genome assembly

The previous release supports annotation of genetic variation mapped on human genome reference assemblies NCBI36/hg18 and GRCh37/hg19. In addition, the current release supports the latest GRCh38/hg38 release. The query interface has been redesigned with dedicated navigation tabs for the three assemblies. The majority of annotations are available for all assemblies. These include characterizing consequences on the transcriptome/proteome, inferring variant mapping and frequencies from public catalogues of human variation and genotype data, finding overlaps with potential regulatory, structural and conserved elements, and retrieving links with previous disease/phenotype association studies. Due to the unavailability of the primary annotation datasets across all assemblies, two annotation categories remain assembly-specific, i.e., predicting functional impact of non-coding variants (for hg19) and neo-epitope prediction for the immunotherapeutic application (for hg38). These will be added to other assemblies as soon as the primary datasets are made available from their respective sources. A complete list of the annotation categories is provided in Supplementary Table S1.

#### Enriched regulatory data

The last few years have seen a marked increase in human genome research on noncoding regions, which constitute over 98% of the genome and potentially possess millions of regulatory elements and noncoding RNA genes. Several large-scale international collaborative efforts such as the Encyclopedia of DNA Elements (ENCODE) (6), Roadmap Epigenomics Mapping Consortium (http://www.roadmapepigenomics.org/) and the FANTOM project (7) have been releasing data on genome-wide mapping of potential regulatory sequences in the form of transcription factor binding sites (TFBS), CTCF binding sites, promoters, promoter flanking regions, open chromatin regions, enhancers and transcription start sites (TSS). Many of the genetic variants associated with disease/phenotype uncovered by genome-wide association studies (GWAS) are found to be residing within or near these ENCODE and Epigenome defined locations. In the previous release of SNPnexus, users could investigate whether the query variants disrupt any of the prominent regulatory elements including TFBS, small regulatory RNAs, miRNAs, putative miRNA target sites, predicted 5′-terminal exons/promoters, CpG islands or VISTA enhancers. The current release has integrated rich resources on regulatory elements from ENCODE and Roadmap Epigenomics as well as Ensembl Regulatory Build – a database of coordinated regulatory annotation of the genome (8). By bringing together all these resources, SNPnexus provides the most extensive information on the putative regulatory impact of the variants.

#### Functional impact prediction for noncoding variants

The vast majority of germline and somatic variations occur in the noncoding part of the genome, only a small fraction of which are believed to be functional. From the tens of thousands of noncoding variations detectable in each genome, distinguishing the potential functional variants from non-functional ones is a challenge. Going beyond SIFT and PolyPhen predictions for deleterious effect of coding variants on protein functions, SNPnexus users can now obtain the predicted functional impact of noncoding variants from eight popular noncoding variant scoring algorithms, namely CADD (9), DeepSEA (10), Eigen (11), FATHMM-MKL (12), fitCons (13), FunSeq2 (14), GWAVA (15) and ReMM (16). Each of these systems uses diverse criteria and computational methods to provide a simple continuous functional score for noncoding variants/regions, placing these within the spectrum of being non-functional and functional (Supplementary Table S2). CADD, GWAVA, FATHMM-MKL and ReMM integrate a range of annotations such as regulatory features, conservation metrics, genic context and genome-wide properties to derive classifiers that differentiate disease-associated/deleterious variants from benign/neutral variants. DeepSEA learns regulatory sequence codes directly from large-scale chromatin-profiling data generated from ENCODE, while fitCons estimates the selective pressure for functionally important genomic regions based on polymorphism and divergence. FunSeq2 and Eigen separate functional and non-functional variants through a weighted scoring system that combines the relative importance of various annotation features. By enabling a single access to these multiple pieces of predictive evidence, SNPnexus now provides a powerful platform to pinpoint the most functionally important and potentially causative noncoding variants.

#### Updated linkage with population and disease association data

Alongside the genotypes and allele frequency data from 11 human populations gathered by the HapMap Project (http://hapmap.ncbi.nlm.nih.gov/), the new release also provides minor allele frequency (MAF) data generated from the 1000 Genomes (1KG) Project (http://www.internationalgenome.org/). 1KG data covers 26 different populations around the globe grouped together in 5 super populations: AFR (African), AMR (Ad Mixed American), EAS (East Asian), EUR (European) and SAS (South Asian). Since the release of Phase 3 data in 2013 with information on over 84 million genetic variants (MAF > 1%), the 1KG project has overshadowed the utility of HapMap data and established itself as a research standard for population genetics. SNPnexus also provides MAF data generated as part of the Exome Aggregation Consortium (ExAC) (17). ExAC contains a catalogue of human genetic diversity generated from exome DNA sequence data for 60 706 unrelated individuals of diverse ancestries, which provides direct evidence for the presence of widespread mutational recurrence.

With ever-increasing number of association studies identifying new relationships among genetic variation and phenotypes, SNPnexus has added another resource ClinVar (18) along with the Genetic Association Database (GAD) (19), the NHGRI genome-wide association study (GWAS) catalogue (20) and the Catalogue Of Somatic Mutations In Cancer (COSMIC) (21) to extract any disease/phenotype-related information about the query variants that have been reported in public domain.

#### Immunotherapeutic application

It has now become well established that somatic variation within a tumour gives rise to neo-epitopes (i.e. small peptides that could be recognized and targeted by T cells) that may make strong candidates for personalized cancer immunotherapy vaccines (22). Assuming the queried variants are somatic, SNPnexus can now assess their potential significance as neo-epitopes by i) building candidate peptides containing the variation, and ii) predicting the peptides’ ability to elicit immunogenicity through multiple leading algorithms, namely MuPeXI (23,24), MHCflurry (BioRxiv: https://doi.org/10.1101/174243) and NetTepi (25). For each non-synonymous coding variant identified using Ensembl annotations, SNPnexus builds 17-mer wild-type and respective mutated peptide pair centred on the position of reference/altered amino acid. If the 17-mer contains a stop codon, it is discarded from the subsequent analysis. Based on the user selection of a neo-epitope peptide of length *n*, each *n*-mer subsequence from the 17-mer mutated peptide is considered as candidate neo-epitope peptide. Binding to a major histocompatibility complex class I (MHC-I) molecule is considered as the essential factor for determining the viability of a peptide as neo-epitope. For user-specified human leukocyte antigen (HLA) allele type corresponding to MHC-I protein, SNPnexus employs NetMHCpan and MHCflurry to report the predicted peptide-MHC (pMHC) binding affinity of each *n*-mer mutated and wild-type peptide pair in nanoMolar (nM) unit. SNPnexus also reports prediction scores from NetTepi, which integrates pMHC binding affinity with pMHC stability and T-cell propensity in its calculations. By reporting scores from multiple immunogenicity prediction methods, SNPnexus enables users to make a better-informed decision on subsequent wet-lab investigations.

### Updated data sources

SNPnexus utilizes primary annotation datasets from different sources to instantly calculate and report variant-centric functional annotations. Currently, we maintain three separate databases for GRCh38/hg38, GRCh37/hg19 and NCBI36/hg18 assemblies. The primary datasets for annotation categories present in the previous release remain unchanged. Most of the primary datasets for hg38 assembly are downloaded from either UCSC genome annotation database or Ensembl 90 data dumps. Among others, the 1KG Project datasets are downloaded from the International Genome Sample Resource (IGSR) FTP site. ExAC, COSMIC and miRBase data are downloaded from their respective FTP sites. The most recent ClinVar data are extracted from the NCBI ClinVar FTP site. Most of the non-coding variant scoring tools except DeepSEA provide pre-computed genome-wide scores, which are downloaded. No such pre-computed scores are available for DeepSEA, for which the respective standalone tool is installed in SNPnexus server and executed for each submission. Similarly, the standalone versions of the three neo-epitope prediction tools are installed and executed from the SNPnexus server. The complete list of data sources for SNPnexus annotation categories is provided in Supplementary Table S3.

### Improved query interface, result presentation and documentation

The query interface has been redesigned with dedicated navigation tabs for different sequence assemblies. It allows users to analyse variants mapped on different assemblies with greater ease. The results are presented with easier navigation facility and in an interactive tabular format with filtering, pagination and sorting options, and are available for download in multiple formats. Where appropriate, results are also summarized in graphical format using the open source visualization library Google Charts. The website contains an *About* section with an overview of the available annotation categories. The *User Guide* compiles the sources of primary annotation data, followed by exhaustive descriptions of query format and output data types. An *Example* section is available, with practical demonstrations of how to use SNPnexus.

## DISCUSSIONS AND FUTURE DIRECTIONS

SNPnexus and other similar resources work on the principle of aggregating/calculating variant annotation information from disparate sources, which would be otherwise laborious for researchers to explore individually. As such, users need to apply their own discretion when interpreting findings reported in SNPnexus, notably those from the prediction algorithms (e.g. functional impact of coding and non-coding variants, putative neo-epitopes) with varying efficiency. Users should consult the individual resources and related peer-reviewed publications to check the reliability of the results generated by these algorithms.

To the best of our knowledge, no single tool, or combinations thereof, available in the public domain provides the range of functionalities offered by SNPnexus. A few command-line-based tools such as ANNOVAR (26), Ensembl Variant Effect Predictor (VEP) (27), SnpEff (28) and VAAST (29) provide a similar breadth of support for functional annotations of both novel and known variants. However, unlike SNPnexus, whose web interface is designed to be used comfortably by biologists, those tools require users to have programming expertise to prepare the underlying databases, install/run the tool on their own machine and parse the output files to interpret the results. wANNOVAR (30; http://wannovar.wglab.org), the freely accessible web version of ANNOVAR, doesn’t provide all the functionalities provided by its command line version. With the provision of predicted functional impact of noncoding variants and putative neo-antigen detection, SNPnexus also surpasses the functionalities offered by the Ensembl VEP Web (http://www.ensembl.org/vep).

The enhancements made in the current release of SNPnexus ensure that it remains a cutting-edge tool and continues to contribute toward maximizing the knowledge extracted from sequence variation data. Looking forward, the analytical focus of SNPnexus will expand toward multi-sample cohort study. This will allow users to build genetic variation profiles within and between samples, highlighting the shared variants and significantly mutated genes contributing to the phenotype studied. Based on the fully annotated variant results in one individual and/or across multiple samples, SNPnexus could then derive a prioritized list of putative disease-associated variants and genes for further investigation of clinical utility. At the same time, the addition of neoepitope prediction feature has propelled SNPnexus to the forefront of personalized medicine with the potential to intersect genomics and immunology. We are planning further useful expansions, for example, mining a candidate neoepitope in known antigen databases and assessing the expression pattern of the corresponding mutated gene, to grow SNPnexus’ application in personalized immunotherapy.

## Supplementary Material

Supplementary Data
